# A mobile health application use among diabetes mellitus patients: a systematic review and meta-analysis

**DOI:** 10.3389/fendo.2024.1481410

**Published:** 2024-10-11

**Authors:** Tesema Etefa Birhanu, Yonas Deressa Guracho, Selamawit Worku Asmare, Diriba Dereje Olana

**Affiliations:** ^1^ Department of Biomedical Science (Clinical Anatomy), Institute of Health, Faculty of Medicine, Jimma University, Jimma, Ethiopia; ^2^ Faculty of Engineering and Information Sciences, University of Wollongong, Wollongong, Australia; ^3^ College of Medical and Health Sciences, Bahir Dar University, Bahir Dar, Ethiopia; ^4^ Department of Dermatology & Venereology, Yekatit-12 Hospital Medical College, College of Medicine, Addis Ababa, Ethiopia; ^5^ Department of Biomedical Science (Medical Physiology), Institute of Health, Faculty of Medicine, Jimma University, Jimma, Ethiopia

**Keywords:** mobile/smartphone, mobile health, app user, interest, and diabetes mellitus patients

## Abstract

**Background:**

Mobile health technologies are increasingly acknowledged as a cost-effective and convenient means of delivering top-notch healthcare services to patients in low- and middle-income countries. This research explores the utilization of mobile health applications in managing, monitoring, and self-care for adult diabetes mellitus (DM) patients. The objective is to gain insight into how diabetic patients currently utilize Mobile health applications for self-management and their inclination to use them in the future.

**Methods:**

The authors conducted a systematic review and meta-analysis using the Preferred Reporting Items for Systematic Reviews and Meta-Analyses (PRISMA) guidelines. They included articles that reported on the use of mobile/smartphone applications for diabetic mellitus disorders, focusing on ownership, application use, future interest in use, and use patterns. The search was conducted in the PubMed, Web of Science, Embase, and SCOPUS electronic databases, with various published articles from January 2016 up to February 2024. The methodological quality was evaluated using the Joanna Briggs Institute critical appraisal tool. Statistical techniques were applied, including the heterogeneity test, publication bias assessment, Egger’s test, and funnel plots. The pooled prevalence was calculated using meta-analysis proportion with a random-effects model.

**Results:**

Thirteen studies were included, out of 4568 recognized articles. The pooled prevalence of mobile health application use for current diabetic management self-management, future interest in using the application for diabetic disorder self-management, and lack of belief in mobile health application users for self-management was 35%, 57%, and 39%, respectively. We observed significant heterogeneity (I^2^ = 97.7, p=<0.001), but no significant publication bias was detected on Egger’s test.

**Conclusions:**

Our meta-analysis results show that over one-third of individuals use mobile health applications for diabetic self-management, and more than half of individuals would like to manage their diabetes mellitus in the future by using mobile health applications. These mobile health apps may be promising in future diabetes mellitus self-management. However, we still need to study the effectiveness of these apps. In addition, adopting mobile health apps based on the cultural context makes this self-management more achievable, practical, and impactful for individuals with diabetes.

**Systematic review registration:**

https://www.crd.york.ac.uk/prospero/, identifier 42024537917.

## Introduction

The utilization of voice calls, text messaging, wireless data transmission, and mobile applications to facilitate healthcare delivery is known as mobile health (mHealth) (1). Digital health encompasses wearable devices, mHealth, health information technology, telehealth, or telemedicine to enhance healthcare accessibility, reduce expenses, remove inefficiencies, and improve patient outcomes (2). Evidence from various sources suggests that mHealth technologies offer advantages to people with diabetes in developing nations. These tools support individuals in better managing their blood sugar levels, fostering adherence to a nutritious diet, promoting increased physical activity, and decreasing the chances of discontinuing medical treatment (3, 4).

Mobile health technologies are increasingly acknowledged as a cost-effective and convenient means of delivering top-notch healthcare services to patients in low- and middle-income countries. These countries often grapple with fragile health systems, a high prevalence of tropical diseases, infectious diseases, and a high mortality rate (5). The potential of this cutting-edge technology is to involve the health system, healthcare professionals, and patients in advancing high-quality healthcare services (6).

Numerous mHealth solutions have emerged, but many need to provide clear evidence of users’ interest, usage level, and overall satisfaction. Conducting this review is necessary to synthesize existing research, identify trends, and pinpoint current gaps for future recommendations. This will enhance our understanding of how mHealth technologies can be effectively integrated into diabetes care, ultimately informing practitioners and developers on optimizing these tools for better patient engagement and health management. The review aims to clarify the discrepancies in findings from previous studies, highlighting the necessity of a unified understanding of how these applications facilitate diabetes management. By systematically analyzing the available data, this study contributes valuable insights into the practical applications of mHealth technology, guiding healthcare providers and researchers in optimizing these tools for diabetic self-management practices. The utilization of mHealth applications by individuals with diabetes mellitus aids in developing personalized interventions for each patient, identifying areas of limited knowledge, and improving evidence-based therapy. There is a scarcity of comprehensive studies with meta-analyses in existing literature that assess the use of mHealth applications in the care, monitoring, and self-management of adult diabetes mellitus patients. Therefore, this current study systematically examines the literature on the use of apps for present diabetes mellitus management, interest in using apps in the future for mental health conditions, and the reasons for not using apps for diabetic self-care.

## Methods

### Data sources and search strategies

The systematic review and meta-analysis adhered to Preferred Reporting Items for Systematic Reviews and Meta-Analyses (PRISMA) guidelines (7). We have registered our meta-analysis in PROSPERO (CRD: 42024537917) to prevent duplication. Our comprehensive search utilized Boolean operators (“OR” and “AND”) to comprehensively explore the PubMed, Web of Science, Embase, and SCOPUS electronic databases. The literature search and screening took place from 05/01/2024 to 30/03/2024. The databases were searched using the following comprehensive terms: “(“Mobile Application” OR “apps” OR “mobile apps” OR “Smartphone application”) AND (“diabetes mellitus care” OR “diabetes mellitus” OR “diabetes mellitus self-care management” OR “DM”) AND (“Patients” OR “client” OR “individual*”).

### Selection of studies

All relevant articles identified through the search strategy were added to Zotero online. The primary author utilized the Zotero duplication check and manager to eliminate any redundant articles to prevent duplication. Each author then individually evaluated the titles and abstracts of the remaining papers. The authors identified studies that aligned with the inclusion criteria and research objectives through this meticulous screening process. The authors collaborated to address cases with differing perspectives through careful deliberation and discussion. After reviewing their titles and abstracts, the chosen papers received a thorough assessment of the entire content. This thorough evaluation ensured that only relevant and successful publications were included in the systematic review and meta-analysis, allowing for a full examination of the articles that were reviewed. The research team aimed to minimize bias and enhance result reliability by employing a well-organized and systematic methodology in the study selection. This comprehensive methodology contributes to the overall validity and robustness of the systematic review and meta-analysis by furnishing pertinent details about the study subject.

### Outcome measurement

Research studies were undertaken to assess the use of mHealth applications for diabetes management, as well as the attitudes of diabetes patients toward these apps. The proportion of diabetes patients using such applications for self-management was calculated by dividing the number of app users by the total number of smartphone users. Similarly, the percentage of patients willing to use mHealth applications for future diabetes self-management was determined by dividing the number of interested individuals by the total number of smartphone owners.

### Eligibility criteria

The evaluation was focused explicitly on studying patients with diabetes mellitus. The inclusion criteria encompassed studies that investigated the percentage of individuals using smartphones, the utilization of mHealth applications for diabetes management, and the willingness of patients to embrace these apps in the future. Only original research meeting these criteria was considered for the evaluation. The analysis was confined to cross-sectional studies published in English from January 2016 to February 2024 to ensure consistency and relevance. The review did not involve the general public or medical professionals. Moreover, unpublished papers, conference presentations, systematic reviews, and thesis papers were excluded from the final analysis.

### Quality assessment

The chosen publications were rigorously assessed by two authors using the critical assessment technique of the Joanna Briggs Institute Meta-analysis of Statistics Assessment and Review Instrument (JBI-MAStARI). In the event of any disagreements, a third author provided a resolution. This approach was employed to collect primary outcome data and to evaluate the methodological rigor and overall quality of the included studies. The review’s objective was to comprehensively assess the selected publications while upholding a high standard of evidence (8).

### Data extraction

The researchers meticulously chose relevant articles using a specific template and entered the gathered data into a Microsoft Excel spreadsheet. The data extraction process encompassed author names, publication year, study location, design, population, average age, sample size, response rate, data collection method, and any pertinent results related to the topic of interest. This systematic data extraction method ensured that all essential information from the included studies was methodically organized and collected, facilitating a comprehensive analysis of the research findings.

### Data synthesis and statistical analysis

The relevant research data was meticulously entered into a Microsoft Excel spreadsheet and then transformed into an event/count format to facilitate meta-analysis. Subsequently, this data was imported into STATA version 18 for in-depth analysis, where the lower and upper boundaries of the confidence interval (LBCI), the standard error, and the prevalence/proportion were computed. The combined prevalence was determined using a statistical method based on the meta-analysis proportion. The I^2^ value was also used to assess the variability of the included studies and choose the appropriate model. This comprehensive approach accounted for the variations among the studies, providing valuable insights into the relevance and overall coherence of the findings (9). The study’s findings were depicted using a forest plot. Heterogeneity levels are typically categorized as low, moderate, and high at 25%, 50%, and 75% of I^2^. A funnel plot was employed to assess publication bias (10).

## Results

**Figure 1:**
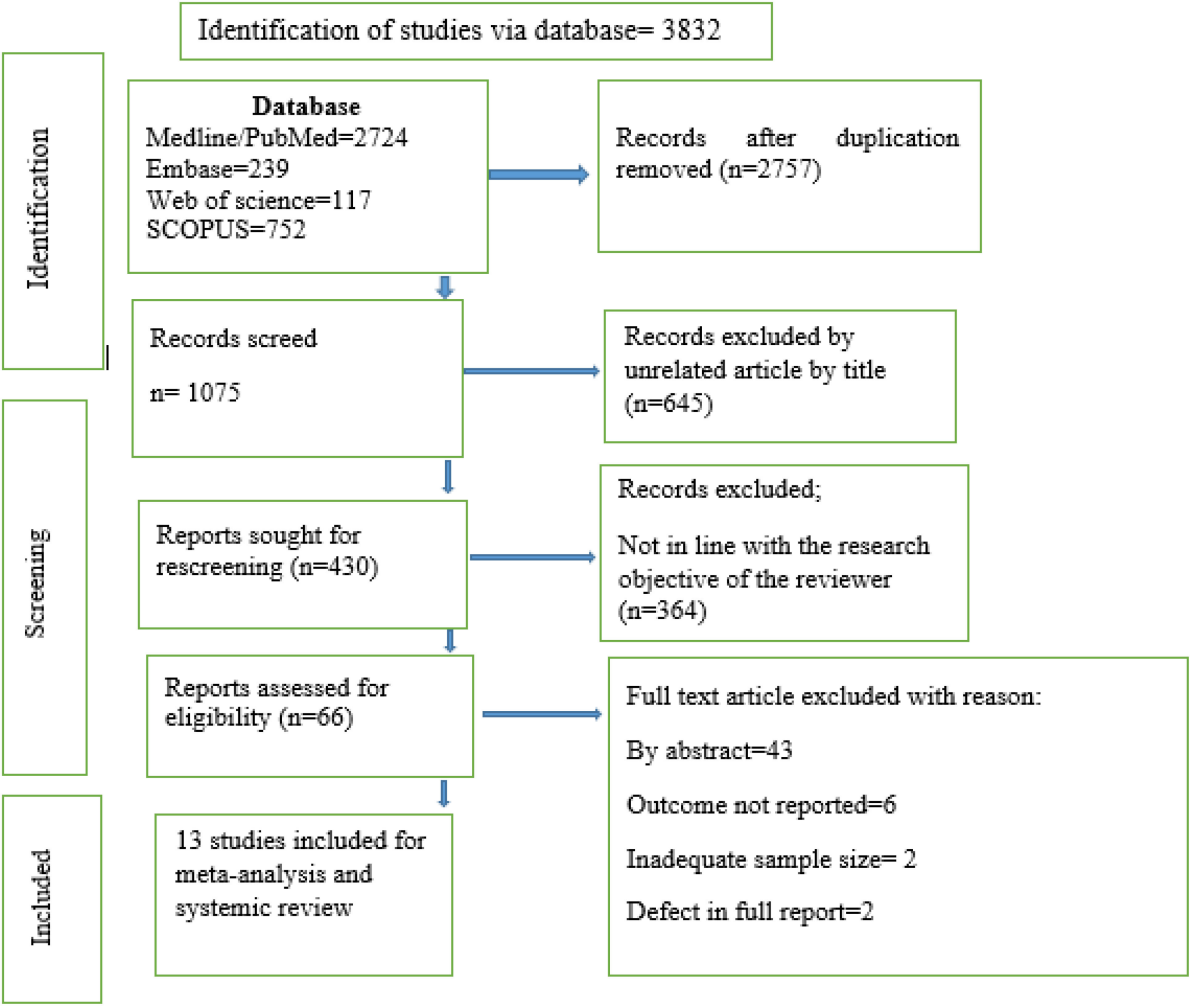
Flow diagram of the included studies in the meta-analysis.

We conducted comprehensive electronic database searches between February 1, 2024, and March 15, 2024. Upon review, 13 studies were identified that met the prescribed criteria for inclusion in the systematic review and subsequent meta-analysis (**Figure 1**).

A total of Thirteen quantitative research studies from various countries, including Australia (n = 2), Europe (n = 4), Asia (n = 3), America (n = 1), the Middle East (n = 2), and online from (Australia, Europe, Asia, and America) one study were included in this meta-analysis and systematic review. These studies covered five continents, reflecting a broad geographical distribution. Twelve of the studies were conducted in specialized diabetic clinics, with participants comprising both inpatients and outpatients. The remaining one research studies focused on a diabetes-related web-based platform. All the studies included in this review were cross-sectional, providing an overview of the prevalence and usage of mHealth applications among people with diabetes. The percentage of people with diabetes using mHealth applications ranged from 14.9% to 54.9%, highlighting variations in prevalence rates across different populations and countries (Refer to **Table 1**).

**Table 1:** Characteristics of the included studies.

1^st^ author and year	Data collection Methods	Study population	Country	Age	Outcomes	Sample	Prevalence	Response rate
Conway et al., 2016 (11)	Web-based or online	DM	Scotland	< and > 50 yrs.	The owner can use their mobile device to choose the mHealth application they desire to use.	142	79(54.9%)	
Trawley et al.2017 (12)	online cross-sectional survey	DM	Australia	18-75yrs.	The app was used to support self-management.	1589	252(14.9%)	
Inagaki et al., 2023 (13)	Internet-based survey,	T2D	Japan	> 18 yrs.	“People with diabetes mellitus used healthcare apps.”	208	79(37.9%)	
Dobson et al., 2017 (14)	Online Phone completing a paper copy of the survey	T2D	New Zealand	16-24yrs	Smartphone apps for diabetes management are helpful for intelligent mobile phone owners.	141	38/115 (33%)	82%
Rangraz et al., 2020 (15)	Self-administrated	T2D	Iran	53.18( ± 15.05) yrs.	They use mobile phones for diabetes self-management and are interested in using apps in the future.	176	95/176(54%)	
Boyle et al., 2017 (16)	Red cap electronic data capture tools	DM patients	New Zealand	50.0 (15.7)yrs.	Utilize apps for better diabetes self-management.	539	37/158 (19.6%)	35%
Adu et al., 2018 (17)	online survey	Both T1D and T2D	Australia, Europe, Asia, and America	18-79 yrs.	Mobile apps for managing diabetes Common self-management techniques for people with diabetes.	217	106(48.8%)	
Trawley et al., 2016 (18)	online survey	TID	Australia	10-19 yrs.	Utilizing an application for managing diabetes	425	87(21%)	
Stühmann et al., 2020 (19)	Interviews	T2D	Germany	18 to 91yrs.	Health app user Determinates of health app use.	481	171(41.1%)	481
Zhang et al., 2019 (20)	Chat using the Web-based survey tool	DM	China	Mean 41.3 yrs.	Diabetic health app user Factor associated with app use.	382	197(51.57%)	
Mehbodniya et al., 2021 (21)	Interview	T2D	India	>18yrs.	mHealth app user user for the future	200	69(34.5%	
Stockman et al.,2019 (22)	A web-based survey	DM,	USA	>18 yrs.	Diabetic health application user	148	29(19.6%)	
Al-Nozha et al., 2022 (23)	A web-based survey	DM	Saudi Arabia	38.2 ± 12.4 yrs.	Awareness of using new technological Mobile health applications use	452	312/61(19.5%)	

The study utilized the JBI Critical Appraisal Tools checklist, which consists of nine items with response options of “Yes,” “No,” or “Not applicable.” Four of the included investigations achieved a perfect score of nine out of nine, while the remaining studies received ratings ranging from six to nine. The meta-analysis incorporated all relevant studies (refer to **Table 2**).

**Table 2:** The Joanna Briggs Institute Critical Appraisal tools (JBI) for included studies in use in JBI Systematic Reviews and meta-analysis.

Quality of domain	Author and year												
	Conway et al., 2016 (11)	Trawley et al., 2017 (12)	Inagaki et al., 2023 (13)	Dobson et al., 2017 (14)	Rangraz et al., 2020 (15)	Boyle et al., 2017 (16)	Adu et al., 2018 (17)	Trawley et al., 2016 (18)	Stühmann et al., 2020 (19)	Zhang et al., 2019 (20)	Mehbodniya et al., 2021 (21)	Stockman et al., 2019 (22)	Nozha et al., 2022 (23)
Q1. Was the sample frame appropriate to address the target population?	Y	Y	Y	Y	Y	Y	Y	Y	Y	Y	Y	Y	Y
Q2. Were study participants sampled appropriately?	Y	Y	NA	Y	Y	Y	Y	Y	Y	Y	Y	Y	Y
Q3. Was the sample size adequate?	Y	Y	N	Y	Y	Y	Y	Y	Y	Y	Y	Y	Y
Q4. Were the study subjects and the setting described in detail?	Y	Y	Y	Y	Y	Y	Y	Y	Y	Y	Y	Y	Y
Q5. Was the data analysis conducted with sufficient coverage of the identified sample?	Y	Y	N	NA	Y	Y	Y	Y	Y	Y	Y	Y	Y
Q6. Were valid methods used for the identification of the condition?	NA	Y	Y	Y	Y	Y	Y	N	Y	Y	Y	N	N
Q7. Was the condition measured in a standard, reliable way for all participants?	Y	Y	Y	Y	Y	Y	Y	NA	Y	NA	Y	Y	N
Q8. Was there an appropriate statistical analysis?	Y	Y	Y	Y	Y	Y	Y	Y	Y	Y	Y	Y	Y
Q9. Was the response rate adequate, and if not, was the low response rate managed appropriately	NA	N	Y	Y	NA	Y	Y	N	Y	Y	Y	Y	Y
Total score out of 9	7	8	6	8	8	9	9	6	9	8	9	8	8

NA, Not applicable.

### mHealth applications user and interest in using the application in the future for diabetic self-management

As per the findings of this systematic review and meta-analysis, individuals with diabetes currently use mHealth applications and express an interest in utilizing them to manage their condition in the future. Out of the studies included, 13 indicated that participants were using mHealth applications, three expressed willingness to use these apps in the future for diabetes self-management, and the remaining three exhibited skepticism towards the use of mHealth applications.

The research revealed that 14.9% to 54.9% of participants across the studies reported current usage of mHealth applications. The pooled overall prevalence of mHealth application usage for diabetic self-management in this systematic review and meta-analysis was determined to be 35% (95% CI: 26%–44%). In this systematic review and meta-analysis, the overall pooled prevalence of individuals interested in using the mHealth application for their diabetic self-management in the future was 57% (95% CI: 46%-68%). In this systematic review and meta-analysis, the overall pooled prevalence of lacking belief in mHealth applications for diabetic self-management was 39% (95% CI: 34%-43%) (Refer to **Table 3**).

**Table 3:** mHealth application users, interest in future use, and skepticism about mHealth application studies.

mHealth app use	Number of studies	Pooled prevalence	95%CI	I^2^	P-value
Current users	13	0.35	0.26-0.44	97.74%	0.00
Interest to use	3	0.57	0.46-0.68	83.91%	0.00
Not belief in mHealth application use	3	0.39	0.34-0.43	68.25%	0.08

**Figure 2:**
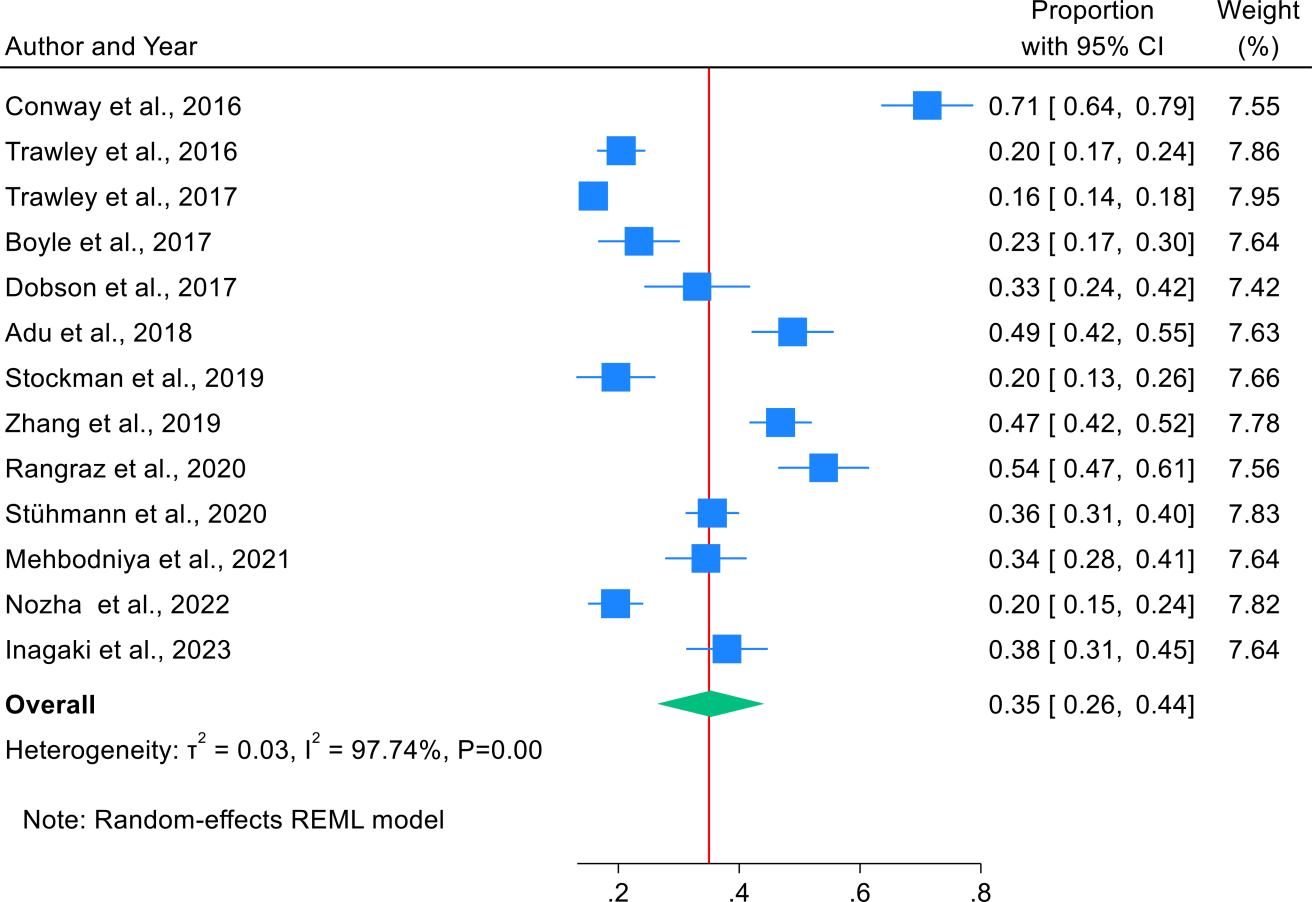
Forest plot showing the combined prevalence of study participants using mHealth applications for diabetic self-management.

According to the frost plot, the pooled prevalence of individual studies, as shown in **Figure 2**, ranged from 16% to 71%.

### Handling heterogeneity

**Figure 3:**
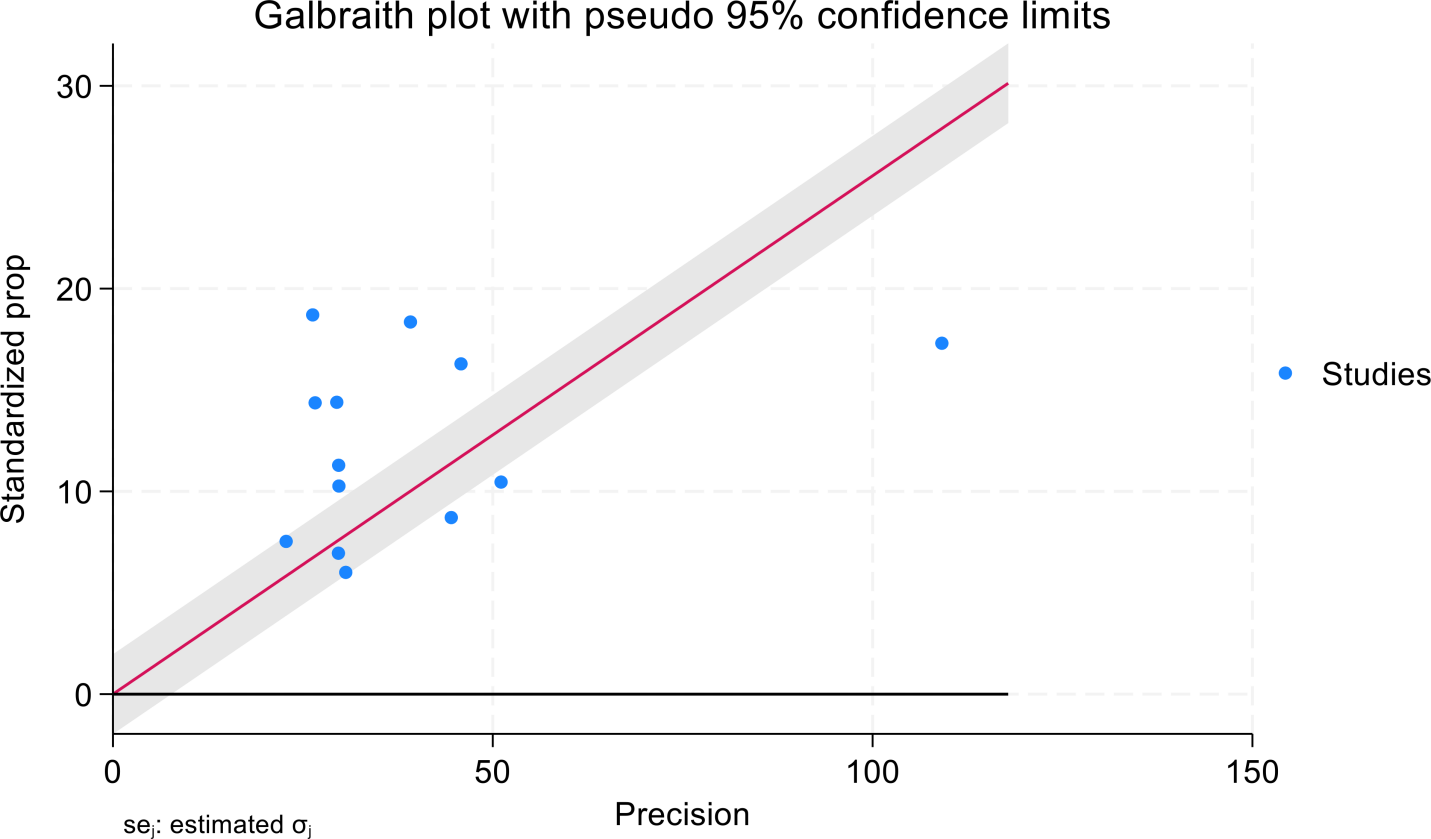
Galbraith plot: visuals of the heterogeneity for meta-analysis of the studies participants of mHealth applications use for their diabetic self-management.

Significant heterogeneity was observed in the random effects model pooled estimate. To address this heterogeneity, sensitivity analysis, and subgroup analysis were performed. We performed a sensitivity analysis to detect potential outliers, no study was found to have an extreme effect on the results (**Figure 3**).

### Publication bias

**Figure 4:**
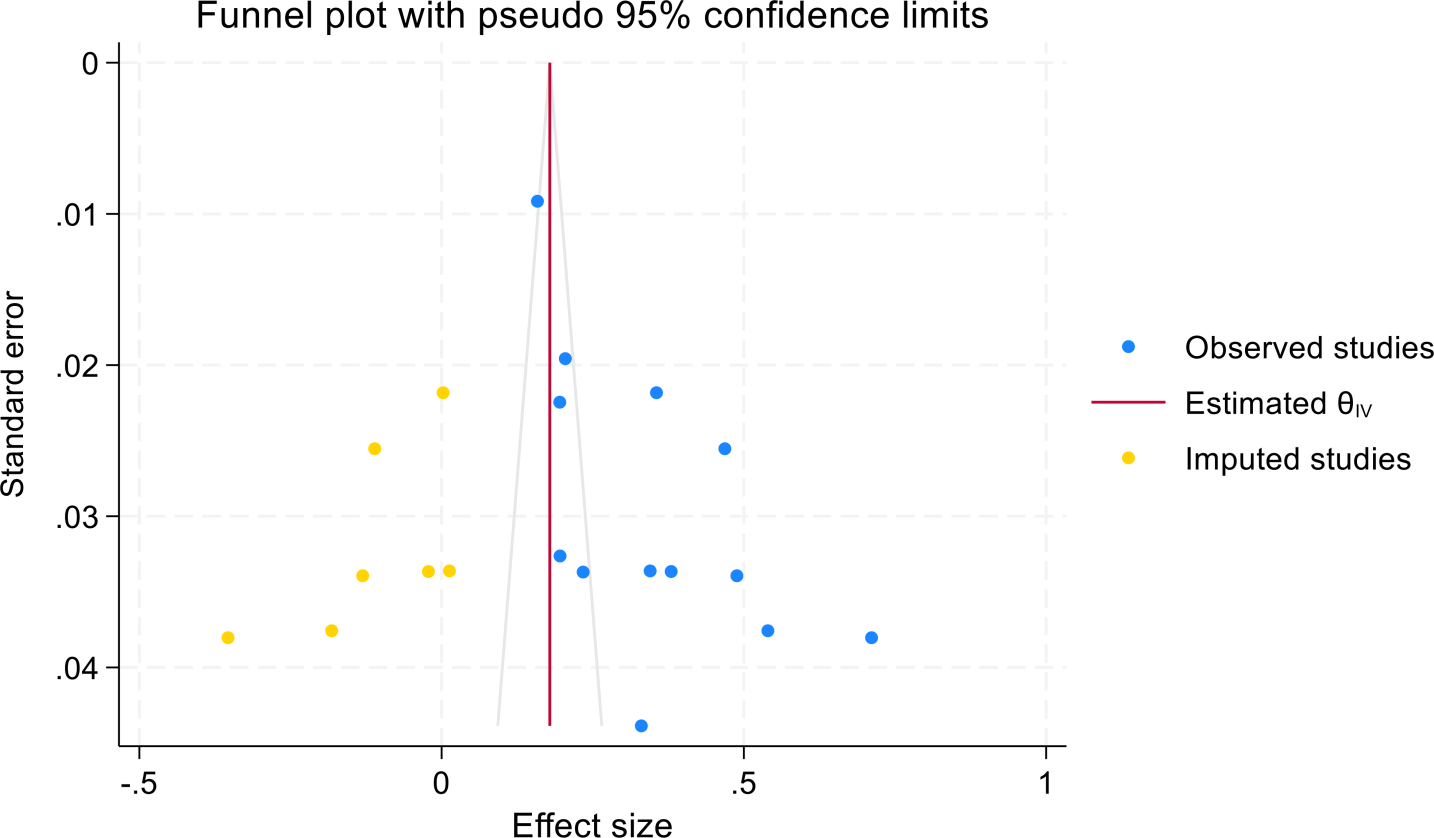
Funnel plot for assessing publication bias in meta-analysis of mHealth application use for diabetic self-management.

**Figure 4** shows a funnel plot to assess publication bias. The trim-and-fill process was used to correct funnel plot asymmetry and estimate potential publication bias. Asymmetric funnel plot indicated publication bias might exist.

### Subgroup analysis

**Figure 5:**
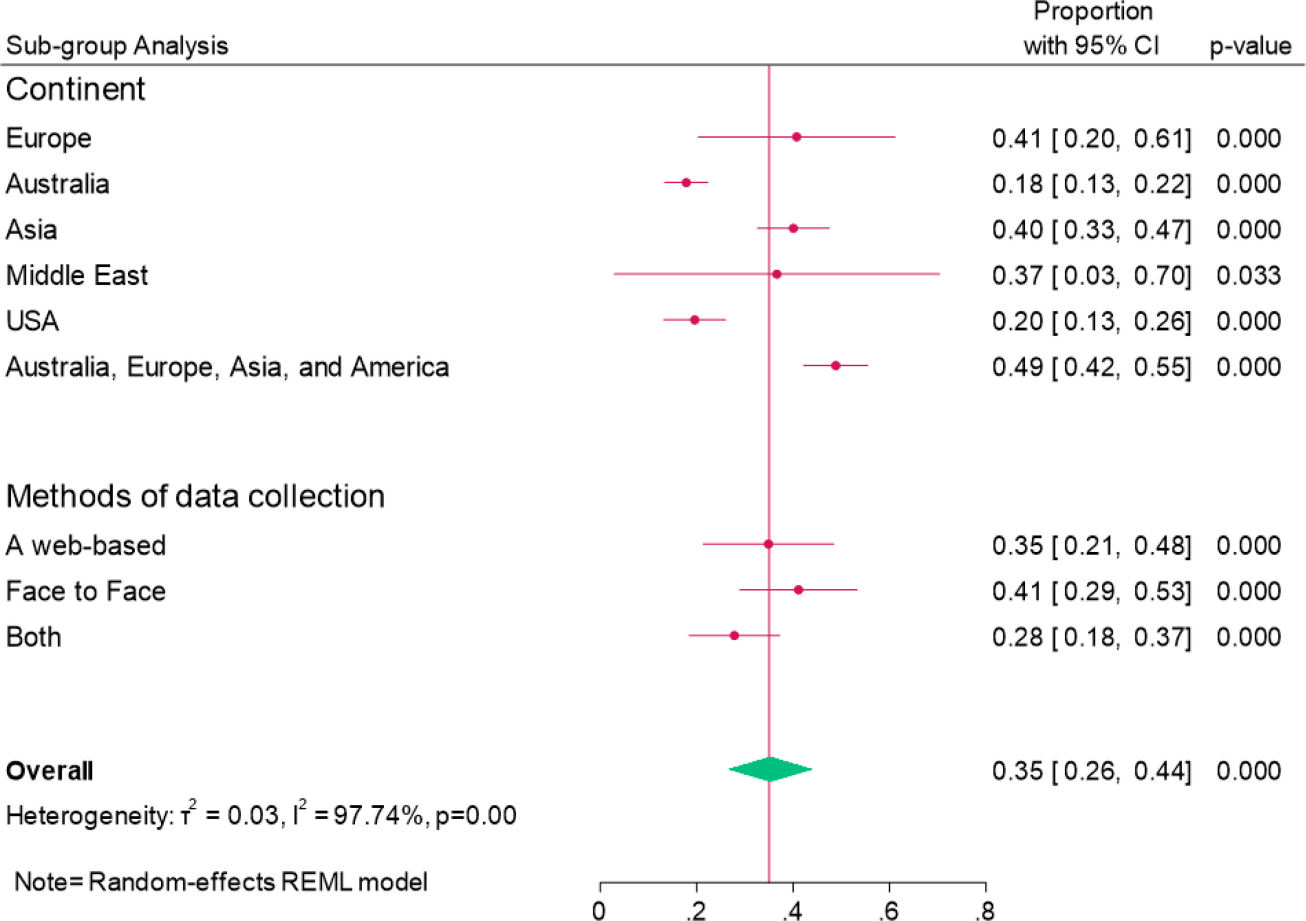
Forest plot showing pooled prevalence of Sub-group analysis of mHealth application users for diabetic mellitus self-management.

The study considered the diversity among mHealth application users and conducted subgroup analysis based on geographical location and data collection method for diabetic self-management. The results revealed that 41% of mHealth application users were in Europe, 18% in Australia, 40% in Asia, and 37% in the Middle East. Furthermore, 35% of participants were surveyed through a web-based method, 41% were surveyed face-to-face, and 28% were part of both methods (web-based and face-to-face) (shown in **Figure 5**).

## Discussion

We have not found any previous comprehensive evaluations or analyses regarding the utilization of mHealth applications by people with diabetes. This research has been registered in the Prospero International Prospective Register of Systematic Reviews with the ID: 42024537917. We identified 13 articles for a systematic review and meta-analysis. Our evaluation focused on the overall prevalence of mHealth application usage and the participants’ interest in using mHealth apps to manage diabetes self-care. Additionally, we conducted subgroup analyses based on the continent and data collection methods.

The systematic review and meta-analysis conducted in the study provide valuable insights into using mHealth applications among individuals with diabetes. The findings indicate that 35% of participants presently use mHealth applications for managing their diabetes. These findings are consistent with the results of a prior study by Bults et al. (24) Anderson et al. (25) Shan R et al. (26),Doupis et al. (27), and Dobson and Hall (28)., who reported that mHealth application use was associated with improved diabetic self-management. These results was also consistent with a study conducted among chronic heart failure (36%) (29). This variability suggests that data collection methods and demographic, cultural, and geographic factors may influence the adoption and utilization of these technologies.

However, the finding of this meta-analysis was lower than the previous study conducted among patients with other health conditions such as hepatitis B(50%) (30), and chronic medical condition(45.8%) (31). Higher than the study conducted on sexual health behavior (29%) (32), liver disease (32%) (33), and mental health disorders (23.29%) (34). The variations in outcomes could be attributed to factors such as the age range of the research participants, the particular study environment, and limited familiarity with mHealth applications. This suggests that it is essential to consider local contexts when assessing and advocating the use of these technologies. The findings indicate that using mHealth applications for diabetes self-care can enhance patients’ self-assurance, aid in monitoring their dietary and physical activity patterns, and improve glycemic control, food intake monitoring, medication management, blood glucose regulation, and communication between patients and healthcare professionals (16, 35–38).

The meta-analysis revealed that a considerable portion of the diabetes population (57%) indicated enthusiasm for utilizing mHealth applications for diabetic self-care managements in the feature. This finding is consistent with a study conducted by Shibutal et al. (39), Humble et al. (40), Skrovseth et al. (41), and Jemere et al. (42), individuals with a specific interest in using mHealth applications to manage their diabetes mellitus in the future. It was also discovered that individuals with diabetes are highly interested in and willing to utilize mHealth applications to manage their condition better. This interest was lower than the study carried out on other health conditions, such as sexual health behavior (67%) (32), and Higher than study conducted among cancer patients(39%) (43). The combined prevalence of skepticism towards mHealth applications for managing diabetes was 39% (95%CI: 34%-43%). This finding aligns with the research conducted by Chahal et al. (44), Faraj et al. (45), and Nundy et al. (46).

Furthermore, subgroup analysis indicates that the highest prevalence of mHealth application usage was observed in Europe (41%), followed by Asia (40%), Australia (18%), and the Middle East (37%). Geographical disparities may reflect differences in healthcare infrastructure and cultural attitudes toward access to mobile health resources, which can affect patient engagement and perceptions of mobile health applications. Additionally, the diversity in data collection methods can introduce variability in the quality and reliability of the data. Because of this heterogeneity, we carefully interpreted our results, highlighting the need for tailored approaches in mobile health interventions that consider local contexts and varying patient needs. By addressing these factors, we can provide more nuanced recommendations for enhancing the adoption and effectiveness of mobile health applications among diabetes patients across different regions.

mHealth applications enhance the ability of patients with chronic illnesses to take care of themselves at an advanced level (47). Research has shown a direct correlation between the utilization of mHealth applications and personal health records. Integrating personal health records through patient portals empowers individuals to proactively manage their health by keeping track of their well-being, monitoring their advancement towards health objectives, maintaining their health between medical appointments, scheduling clinic visits electronically, and enhancing digital communication between patients and healthcare professionals (48–50).

The findings point to the increasing importance of mobile health technologies in diabetes management. Healthcare professionals, policymakers, and app developers need to understand usage patterns in detail and adapt their strategies as the market for mHealth applications expands. By leveraging the widespread interest in and current usage of mHealth apps, stakeholders can devise and implement effective strategies to enhance the integration of these technologies into diabetes self-management practices across different demographics and contexts.

### Limitations

There are some limitations existed in the present study. Firstly, we found significant heterogeneity existed, to explore the source of heterogeneity, we do the sub group analysis. Secondly, the studies were only conducted in developed countries such as the USA, Asia, Europe, Middle East, and Australia, which limits the applicability of the findings globally. Thirdly there’s no studies specifically undertaken in developing countries. Fourthly, since we included the cross-sectional studies, the design of this observational study prevents us from making conclusions about causality. Therefore, it’s essential to interpret the results with caution.

### Conclusion

Our meta-analysis results show that over one-third of individuals use mobile health applications for diabetic self-management, and more than half of individuals would like to manage their diabetes mellitus in the future by using mobile health applications. These mobile health apps may be promising in future diabetes mellitus self-management. However, we still need to study the effectiveness of these apps. In addition, adopting mobile health apps based on the cultural context makes this self-management more achievable, practical, and impactful for individuals with diabetes. Finally, we also recommend that future study focus on the awareness creation, designing mHealth application, and challenges of implementing mHealth in developing countries.

## Data Availability

The original contributions presented in the study are included in the article/supplementary material. Further inquiries can be directed to the corresponding author.
